# Characterization of erythrose reductases from filamentous fungi

**DOI:** 10.1186/2191-0855-3-43

**Published:** 2013-08-08

**Authors:** Birgit Jovanović, Robert L Mach, Astrid R Mach-Aigner

**Affiliations:** 1 Department for Biotechnology and Microbiology, Institute of Chemical Engineering, Vienna University of Technology, Gumpendorfer Str. 1a, Wien A-1060, Austria

**Keywords:** *Trichoderma reesei*, *Aspergillus niger*, *Fusarium graminearum*, Erythrose reductase, Erythritol

## Abstract

Proteins with putative erythrose reductase activity have been identified in the filamentous fungi *Trichoderma reesei*, *Aspergillus niger,* and *Fusarium graminearum* by in silico analysis. The proteins found in *T. reesei* and *A. niger* had earlier been characterized as glycerol dehydrogenase and aldehyde reductase, respectively. Corresponding genes from all three fungi were cloned, heterologously expressed in *Escherichia coli,* and purified. Subsequently, they were used to establish optimal enzyme assay conditions. All three enzymes strictly require NADPH as cofactor, whereas with NADH no activity could be observed. The enzymatic characterization of the three enzymes using ten substrates revealed high substrate specificity and activity with D-erythrose and D-threose. The enzymes from *T. reesei* and *A. niger* herein showed comparable activities, whereas the one from *F. graminearum* reached only about a tenth of it for all tested substrates. In order to proof in vivo the proposed enzyme function, we overexpressed the erythrose reductase-encoding gene in *T. reesei.* An increased production of erythritol by the recombinant strain compared to the parental strain could be detected.

## Introduction

Erythritol is a four-carbon sugar alcohol, which is applied as flavour enhancer, formulation aid, humectants, stabilizer, thickener, and as low-calorie sweetener, of which the latter is the main utilization. It has a natural occurrence in several foods including beer, sake, wine, soy sauce, water melon, pear and grape (O’Donnell and Kearsley [2012]; Sreenath and Venkatesh [2008]) and is well tolerated by the human body (Munro et al. [1998]). Erythritol can be chemically synthesized from dialdehyde starch with a nickel catalyst at high temperatures, but this process is not stereospecific and low in yield, and therefore, not industrialized (Moon et al. [2010]). Instead erythritol is produced in biotechnological processes using osmophilic yeasts obtained by random mutagenesis as *Aureobasidium* sp. (Ishizuka et al. [1989]; Sasaki et al. [1990]), *Trichosporonoides* sp. (Suh et al. [1999]), (*Torula* sp. Oh et al. [2001]), and *Candida magnoliae* (Koh et al. [2003]; Ryu et al. [2000]). As substrate a highly concentrated glucose solution (typically 40% (w/v)) is applied, which is gained from chemically and enzymatically hydrolyzed wheat- and cornstarch. It serves as carbon source and causes high osmotic pressure, which pushes the yeast to produce the osmolyte erythritol (reviewed by (Moon et al. [2010])).

Even though the production of erythritol and the according enzyme, erythrose reductase, have been well studied in yeasts, no such enzymes have yet been identified in filamentous fungi. For this study the filamentous ascomycota *Trichoderma reesei* (telemorph *Hypocrea jecorina*, (Kuhls et al. [1996])), *Aspergillus niger,* and *Fusarium graminearum* (telemorph *Gibberella zeae*) were chosen because of their great importance in biotechnology. The (hemi)cellulases of *T. reesei* are widely used in pulp and paper production (Buchert et al. [1998]; Noé P. [1986]; Welt [1995]), food and feed industry (Galante [1993]; Lanzarini [1989]; Walsh et al. [1993]), textile industry (Koo [1994]; Kumar [1994]; Pedersen [1992]), and more recently, for 2^nd^ generation biofuel (cellulose ethanol) production (Hahn-Hägerdal et al. [2006]; Himmel et al. [2007]; Ragauskas et al. [2006]). *A. niger* is used for the production of organic acids, as citric acid and gluconic acid (Ruijter et al. [2002]), for heterologous protein expression Archer and Turner ([2006]), as well as production of pectinases Bussink et al. ([1992]; Delgado et al. [1992]; Parenicová et al. [2000]) and hemicellulases, such as xylanases and arabinases Gielkens et al. ([1997]; van Peij et al. [1997]). *F. graminearum* is a well studied filamentous fungus because of its relevance as plant pathogen that can infect numerous plants like cereals, but also dicotyledons (Pirgozliev et al. [2003]; Urban et al. [2002]). Additionally, it is also used in biotechnological applications such as heterologous protein expression (Royer et al. [1995]).

In contrast to yeasts, the use of filamentous fungi offers the interesting perspective of using non-food plant biomass (e.g. lignocellulose) as substrate. By secretion of xylanolytic enzymes, these fungi are capable of degrading xylans into their major monomers D-xylose and L-arabinose. They can be directly metabolized to D-xylose-5-phosphate to supplement the pentose phosphate pathway (PPP), from which erythritol is a side product. D-xylulose-5-phosphate and D-ribose-5-phosphate are transferred by a transketolase to D-sedoheptulose-7-phosphate and D-glyceraldehyde-3-phosphate, which are further processed by a transaldolase to fructose-6-phosphate and D-erythrose-4-phosphate. A schematic drawing of the according pathway is given in Additional file 1. Erythritol is formed by de-phosphorylation of D-erythrose-4-phosphate and the following reduction:

D−Erythrose+NADPH→ReductaseErythritol+NADP+

The characterization of the enzyme performing this reduction, namely erythrose reductase, has been done for some yeasts e. g. by (Lee et al. ([2010]), (Lee et al. [2003]), (Ookura et al. [2005])), but until now no such enzyme has been identified for the above-mentioned filamentous fungi.

In this study, we identified by in silico analysis proteins in *T. reesei*, *A. niger*, and *F. graminearum* exhibiting a high sequence similarity to the erythrose reductase (ER1) from *Trichosporonoides megachiliensis*. Accordingly, in this manuscript the corresponding proteins from the three organisms are referred to the term Err1 (Erythrose reductase 1) for easier reading. The respective genes were cloned and their protein products were heterologously expressed and purified. All three putative Err1 proteins were characterized in enzymatic assays with respect to their substrate specificity to D-erythrose and nine other potential substrates. In order to do this, the optimal assay conditions (temperature and pH) for all three enzymes were determined before, and then their usages of the different substrates were tested. Finally, we aimed to prove the function of the putative erythrose reductase in vivo. Therefore, the corresponding *T. reesei* enzyme was overexpressed in this fungus and the production of erythritol in the recombinant strain was compared to the parental strain.

## Materials and methods

### Strains and cultivation conditions

The *T. reesei* strain QM6aΔtmus53 (Steiger et al. [2011]), the *A. niger* strain N400 (CBS 120.49), and the *F. graminearum* strain PH1 (NRRL31084) were maintained on malt extract (MEX) agar, complete medium agar (Pontecorvo et al. [1953]), and small nutrient agar (Brunner et al. [2007]), respectively. The recombinant *T. reesei* strain PEC1, produced during this study, was maintained on MEX agar containing hygromycin B.

Cultivation in shakeflasks was performed in 1-l-Erlenmeyer flasks containing 250 ml (Mandels-Andreotti (MA) medium Mandels [1985]) supplemented with 1% (w/v) D-xylose. For inoculation 10^9^ conida per litre were used. Growth conditions were pH 5, 30°C, and 160 rpm shaking rate. For harvesting mycelia, samples of 60 ml were drawn after 24 h and 30 h. For short-term storage, mycelia were shock-frozen and kept in liquid nitrogen.

### Plasmid construction

The in silico identified *err1* genes from *T. reesei*, *A. niger,* and *F. graminearum* were amplified from cDNA. The cDNA was generated as described below in the according section. Primers were used to introduce restriction sites adjacent to the gene. Primer sequences are given in Table 1. The PCR products were subcloned into pJET-1.2 (Thermo Scientific, Waltham, MA, USA), using chemically competent *Escherichia coli* TOP 10 (Invitrogen, Life Technologies Ltd, Paisley, UK) for plasmid replication.

**Table 1:** Oligonucleotides used during the study

**Name**	**Sequence (5′ - 3′)**^**a**^	**Usage**
err1_A.nig_BamHI_f	ATATA**GGATCC**ATGTCTCTCGGAAAGAAGGTTACTCTC	pGEX-err1A
err1_A.nig_NotI_r	TATAT**GCGGCCGC**TTAAACAATCACCTTATGACCAGCAGGC	pGEX-err1A
err1_T.ree_BamHI_f	ATATA**GGATCC**ATGTCTTCCGGAAGGACC	pGEX-err1T
err1_T.ree_NotI_r	TATAT**GCGGCCGC**TTACAGCTTGATGACAGCAGTG	pGEX-err1T
err1_F.gra_BamHI_f	ATATA**GGATCC**ATGTCTTTCGGTCGAACTGTCACTC	pGEX-err1F
err1_F.gra_NotI_r	TATAT**GCGGCCGC**TTACAGCTTGAGAACAACCTGGTGG	pGEX-err1F
err1_XbaI_f	ATATA**TCTAGA**ATGTCTTCCGGAAGGACC	Vector construction for fungal transformation
err1_Nsi_r	TATAT**ATGCAT**TTACAGCTTGATGACAGCAGTG	
qerr1_f	CTTTACCATTGAGCACCTCGACG	RT-qPCR *err1*
qerr1_r	GGTCTTGCCCTGCTTCTTGG	RT-qPCR *err1*
qact1_f	TGAGAGCGGTGGTATCCACG	RT-qPCR *act1*
qact1_r	GGTACCACCAGACATGACAATGTTG	RT-qPCR *act1*
qsar1_f	TGGATCGTCAACTGGTTCTACGA	RT-qPCR *sar1*
qsar1_r	GCATGTGTAGCAACGTGGTCTTT	RT-qPCR *sar1*

^a^ restriction enzyme sites are given in bold letters.

For the construction of pGEX-err1T, pGEX-err1A, and pGEX-err1F the *err1* gene was excised from pJET-1.2 by *Eco*RI/*Bam*HI digestion and inserted into pGEX-4T-2 (GE Healthcare Life sciences, Little Chalfont, Buckinghamshire, UK).

For the construction of pBJ-PEC1 the vector pRLM_ex30_ Mach et al. ([1994]), which contains the *hph* gene flanked by the *pki* promoter and the *cbh2* terminator, was used. The *hph* gene was removed by *Nsi*I*/Xba*I digestion and subsequently, *err1,* which was excised from JET-1.2 also by *Nsi*I*/Xba*I digestion, was inserted.

### Protoplast transformation

The protoplast transformation of *T. reesei* was performed as described by (Gruber et al. [1990]). 5 μg of the plasmid pBJ-PEC1 and 1 μg pAN7, which confers hygromycin B resistance (Punt et al. [1987]), were co-transformed into the fungal genome.

### DNA analysis

Fungal genomic DNA was isolated by phenol-chloroform extraction, using a FastPrep®-24 (MP Biomedicals, Santa Ana, CA, USA) for cell disruption. Therefore about 100 mg of mycelia was transfered to 400 μl DNA extraction buffer (0.1 M Tris–HCl pH 8.0, 1.2 M NaCl, 5 mM EDTA) and grounded with glass beads (0.37 g Ø 0.01 – 0.1 mm, 0.25 g Ø 1 mm, 1 piece Ø 3 mm) using the FastPrep. Afterwards, the mixture was immediately put on 65°C, supplemented with 9 μM RNase A, and incubated for 30 min. Then 200 μl of phenol (pH 7.9) and 200 μl of a chloroform-isoamyl alcohol-mixture (25:1) were added, and vigorous mixing followed each addition. Phases were separated by centrifugation (12000 g, 10 min, 4°C) and the aqueous phase was transferred into a new vial. DNA was precipitated by addition of the 0.7-fold volume of isopropanol. After 20 min incubation at room temperature the DNA was separated by centrifugation (20000 g, 20 min, 4°C) and washed with 500 μl ethanol (70%). The air-dried DNA pellet was solubilised in 50 μl Tris–HCl (10 mM, pH 7.5) at 60°C.

### RNA isolation and cDNA synthesis

RNA extraction from fungal mycelia was performed with peqGOLD TriFast™ (peqlab, Erlangen, Germany) according to the manufacturer’s procedure, using a FastPrep®-24 (MP Biomedicals, Santa Ana, CA, USA) for cell disruption. RNA quantity and quality were determined with a NanoDrop 1000 (Thermo Scientific, Waltham, MA, USA). A 260 nm/280 nm ratio of at least 1.8 was stipulated for further sample processing.

cDNA synthesis was performed with RevertAid™ H Minus First Strand cDNA Synthesis Kit (Thermo Scientific, Waltham, MA, USA) according to the manufacturer’s procedure, using 0.5 μg of RNA.

### Transcript analysis

Quantitative PCR (qPCR) analysis was performed in a Rotor-Gene Q cycler (Qiagen, Hilden, Germany). The qPCR amplification mixture had a total volume of 15 μl, containing 7.5 μl 2× IQ SYBR Green Supermix (Bio-Rad Laboratories, Hercules, CA, USA), 100 nM forward and reverse primer, and 2 μl cDNA (diluted 1:100). Primer sequences are given in Table 1. As reference genes *act1* and *sar1* were used (Steiger et al. [2010]). All reactions were performed in triplicates. For each gene a no-template control and a no-amplification control (0.01% SDS added to the reaction mixture) was included in each run. The cycling conditions for *act1* and *err1* comprised 3 min initial denaturation and polymerase activation at 95°C followed by 40 cycles of 15 s at 95°C, 15 s at 59°C, and 15 s at 72 s. For *sar1* different cycling conditions were applied: 3 min initial denaturation and polymerase activation at 95°C followed by 40 cycles of 15 s at 95°C and 120 s at 64 s. PCR efficiency was calculated from the Rotor-Gene Q software. Relative expression levels were calculated using the equation

$$\mathrm{relative}\;\mathrm{transcript}\;\mathrm{ratio}=\;E_{r}^{C\left(r\right)}\cdot \;E_{t}^{-C\left(t\right)}\cdot \;E_{\mathrm{ro}}^{-C\left(\mathrm{ro}\right)}\cdot \;E_{\mathrm{to}}^{C\left(\mathrm{to}\right)}$$

where E is cycling efficiency, C is the threshold cycling number, r is the reference gene, t the target gene and o marks the sample which is taken for normalization Pfaffl ([2001]).

### Glutathione S-transferase (GST): Err1 fusion proteins

GST fusion proteins of the erythrose reductases from *T. reesei*, *A. niger*, and *F. graminearum* were expressed using plasmids pGEX-err1T, pGEX-err1A, and pGEX-err1F, respectively, in *E. coli* BL21(DE3)pLysS (Promega, Madison, WI, USA). The protein expression was done in shakeflasks on lysogeny broth supplemented with 100 μg/ml ampicillin at 37°C and 200 rpm. For induction 0.1 mM isopropyl-β-D-thiogalactopyranoside (IPTG) was added when the culture reached an OD_600_ between 0.7 and 0.8. Cells were harvested 3 h after induction by centrifugation, resuspended in phosphate buffered saline supplemented with 1% Triton X-100, and sonicated using a Sonifier® 250 Cell Disruptor (Branson, Danbury, CT, USA) (power 70%, duty cycle 40%, power for 10 s, pause for 50 s, 10 cycles, on ice). Insoluble compounds were separated by centrifugation (2600 g, 10 min, 4°C). Purification of the proteins was performed using GSTrap™ FF (GE Healthcare Life sciences, Little Chalfont, Buckinghamshire, UK) according to standard procedures. The purified protein solutions were stored at 4°C. There was no considerable loss of activity observed within one month under these storage conditions. The addition of glycerol must be avoided because it has an influence on the enzymatic assay described later.

### SDS-PAGE analysis

For the SDS-PAGE analysis a 10% polyacrylamide gel with a tris-glycine buffer (25 mM Trizma® base (Sigma Aldrich, St. Louis, MO, USA), 1.9 mM glycine, 0.5% SDS) was used. Gel casting and running the gel was done with the Mini-PROTEAN® Tetra Cell system (Bio-Rad Laboratories, Hercules, CA, USA). From all three protein expressions 2 μl of the crude extract, 2 μl of the flow-through, and 12 μl of the wash solution, respectively, were applied on the gel. Of the eluated protein from *A. niger* 2 μl, from *F. graminearium* 12 μl, and from *T. reesei* 1 μl were applied. All the samples were supplemented with 4 μl 4x Laemmli sample buffer (Bio-Rad Laboratories, Hercules, CA, USA), filled up with distilled water to a final volume of 16 μl, and incubated for 10 min at 95°C for denaturation. After denaturation, samples were kept on ice until application on the gel. For protein size estimation 2.5 μl of PageRuler™ Prestained ProteinLadder (Thermo Scientific, Waltham, MA, USA) were used. The electrophoresis was carried out at a constant voltage of 160 V. Staining of the gels was done with PageBlue Protein Staining Solution (Thermo Scientific, Waltham, MA, USA) according to the manufacturers protocol.

### Enzymatic assay

Enzymatic analysis was performed according to a slightly modified, previously by Lee et al. ([2003]) described protocol. The reducing reaction was performed in a total volume of 1 ml containing 50 mM Sorenson’s phosphate buffer (pH 6.5), 160 μM NADPH or NADH, 100 μl purified GST::Err1 fusion protein, and 10 mM substrate. As substrates L-arabinose, dihydroxyacetone (DHA), D-erythrose, D-glucose, L-glyceraldehyde, glyoxal, methylglyoxal, D-threose, D-xylose, and D-xylulose were used. In a spectrophotometer the consumption of NADPH or NADH over time was followed at 340 nm at the indicated temperature. After 1 min incubation without substrate the reaction was started by adding 100 μl 100 mM substrate.

The oxidizing reaction was performed in a total volume of 1 ml containing 50 mM Tris/HCl (pH 9.0), 400 μM NADP^+^, 200 μl purified GST::Err1 fusion protein, and 10 mM erythritol. In a spectrophotometer the formation of NADPH over time was followed at 340 nm at a temperature of 40°C. After 1 min incubation without substrate the reaction was started by adding 100 μl 100 mM erythritol.

Enzymatic assays were performed in triplicates. Activity is defined in katal (kat), and 1 katal is the conversion of 1 mol substrate per second. The specific activity k_cat_ is defined as 1 katal per mol enzyme and the catalytic efficacy is defined as k_cat_/K_m_.

### Gas chromatography (GC) analysis

Mycelia were ground under liquid nitrogen. The powder was suspended in 3 ml distilled water and sonicated using a Sonifier® 250 Cell Disruptor (Branson, Danbury, CT, USA) (power 70%, duty cycle 40%, power for 3 min, on ice). Insoluble compounds were separated by centrifugation (20000 g, 10 min, 4°C). Sample preparation for GC was done in triplicates as follows: 300 μl of the supernatant, supplemented with 10 ng sorbitol as internal standard, was gently mixed with 1.2 ml ethanol (96%) and incubated for 30 min at room temperature for protein precipitation. The precipitant was separated by centrifugation (20000 g, 10 min, 4°C). Samples were dried under vacuum and thereafter silylated (50 μl pyridine, 250 μl hexamethyldisilazane, 120 μl trimethylsilyl chloride). For quantitative erythritol determination a GC equipment (Agilent Technologies, Santa Clara, CA, USA) with a HP-5-column (30 m, inner diameter 0.32 mm, film 0.26 μm) (Agilent Technologies, Santa Clara, CA, USA) was used. The mobile phase consisted of helium with a flow of 1.4 l/min, the column temperature was as follows: 150°C for 1 min, ramping 150 – 220°C (ΔT 4°C/min), ramping 220–320°C (ΔT 20°C/min), 320°C for 6.5 min. Detection was performed with FID at 300°C. The retention times were determined using pure standard substances.

## Results

### Identification of putative erythrose reductase proteins by in silico analysis

**Figure 1:**
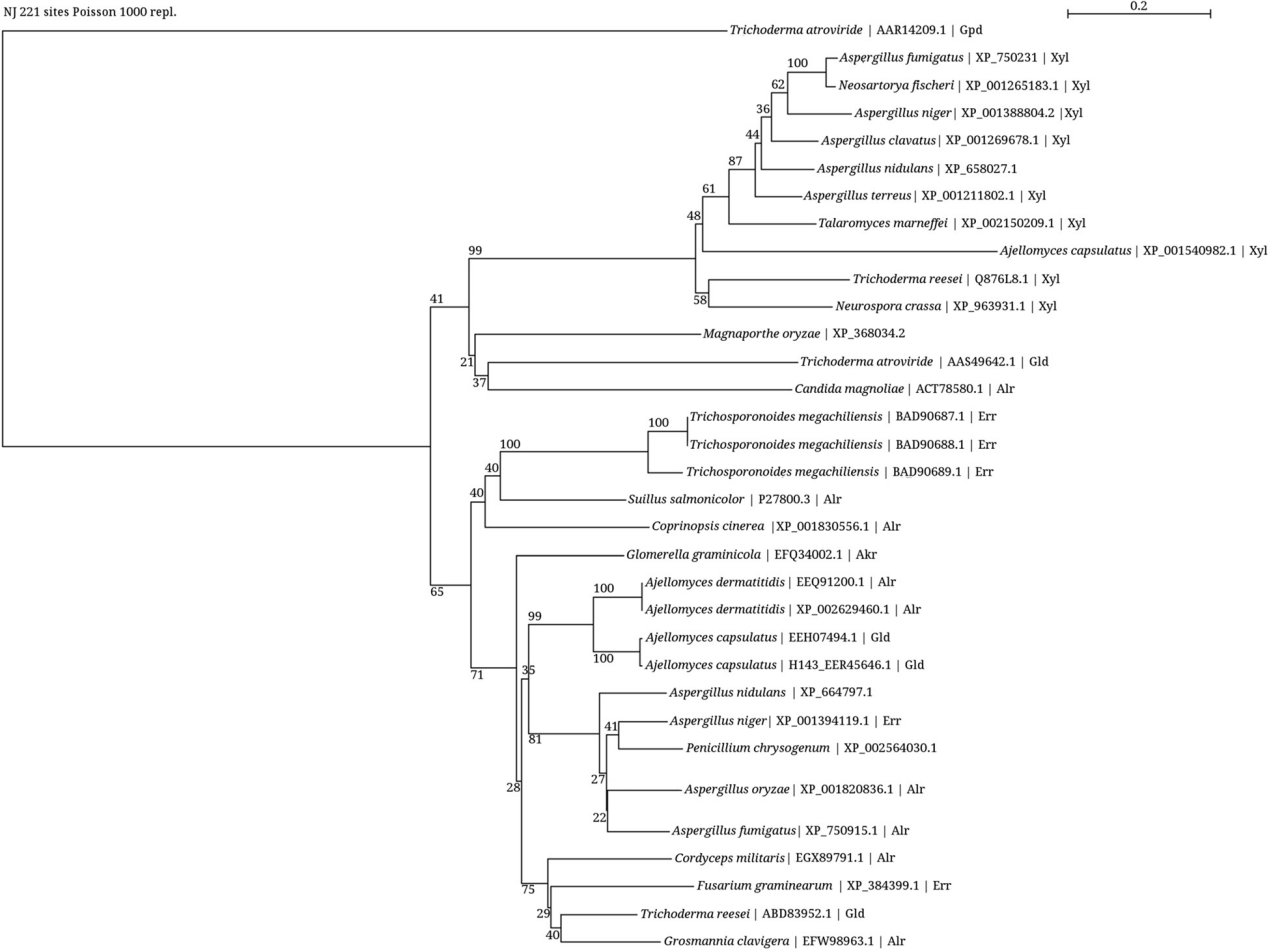
**Phylogram of proteins from the aldo-keto reductase family from various fungi, identified by GenBank accession numbers.** Functional descriptions are given, as far as available, by the abbreviations Akr (aldo-keto reductase), Alr (aldose reductase), Err (erythrose reductase), Gld (glycerol dehydrogenase), Xyl (xylose reductase). Protein sequences were aligned using the seaview program applying the muscle algorithm. The phylogram was constructed in seaview with the neighborhood joining method. Bootstrap was done with 1000 replicates, according bootstrap values are given on the branches.

Ookura et al. ([2005]) biochemically characterized three isoenzymes of the erythrose reductase (ER1, ER2, and ER3) from the industrial erythritol production strain *Trichosporonoides megachiliensis* SNG-42. The protein sequences of ER1 (NCBI accession number BAD90687.1), ER2 (NCBI accession number BAD90688.1), and ER3 (NCBI accession number BAD90689.1) were compared with the NCBI database using BLASTP to find proteins with similar sequence in the filamentous fungi *T. reesei*, *A. niger,* and *F. graminearum*. The following proteins were found in these organisms: for *T. reesei* the NADP-dependent glycerol dehydrogenase (GLD1) (NCBI accession number ABD83952.1, query coverage 98%, max. ident. 50%, E-value 5e-87); for *A. niger* the aldehyde reductase 1 (Alr1) CBS 513.88 (NCBI accession number XP_001394119.2, query coverage 98%, max. ident. 49%, E-value 8e-88); and for *F. graminearum* a hypothetical protein FG04223.1 (NCBI accession number XP_384399.1, query coverage 98%, max. ident. 48%, E-value 1e-98). Query results are given relative to ER3, which showed a slightly better match with the protein found for *T. reesei* than ER1 and ER2. Figure 1 shows the phylogram of the above-mentioned protein sequences. The protein found for *T. reesei* has originally been described as glycerol dehydrogenase Liepins et al. ([2006]), but was not tested with D-erythrose or erythritol as substrate. So the high sequence similarity to ER3 led us to the assumption that this protein might have an erythrose reductase activity. The corresponding enzyme from *A. niger,* Alr1, was only generally recognized as a NADPH-dependent member of the aldo-keto reductase superfamily, but no physiological function was identified up to now. For the *F. graminearum* protein no function was proposed so far.

### Purification of heterologously expressed Err1 proteins

**Figure 2:**
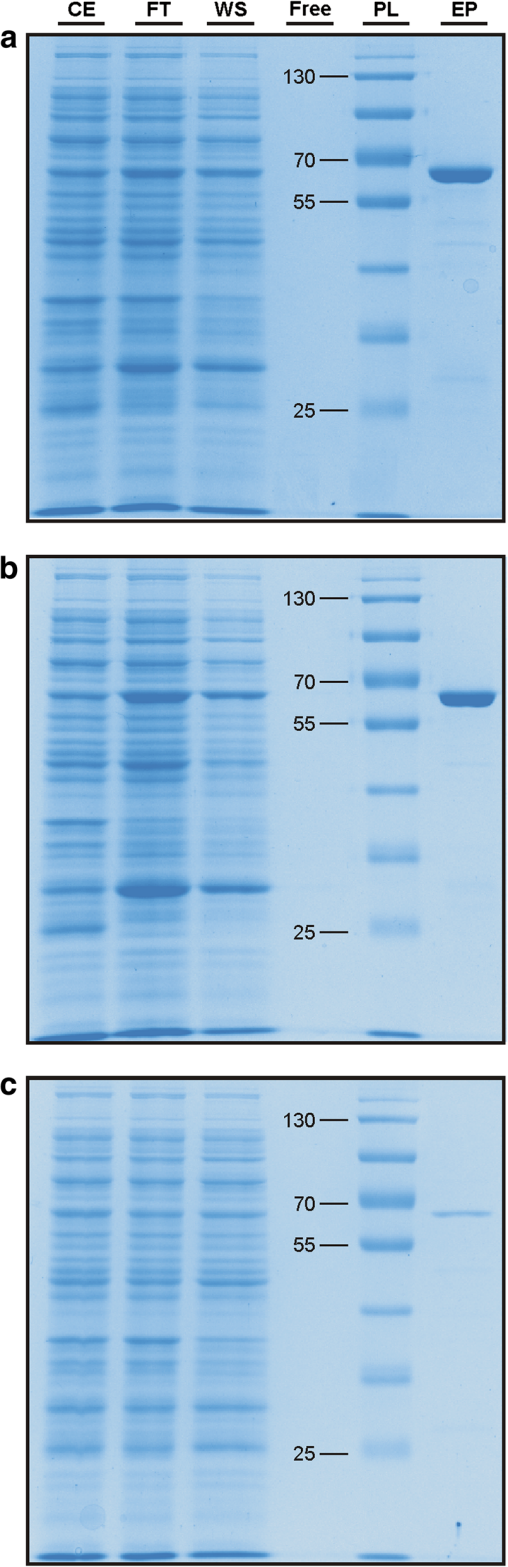
**SDS-PAGE analyses of the purification of the three GST:Err1 fusion proteins.** Applied were the crude extract (CE), the flow-through from application of the crude extract (FT), the wash solution (WS) and the eluated protein (EP) resulting from expression of GST proteins fused to the Err1 from *T. reesei***(a)**, *A. niger***(b)**, and *F. graminearum***(c)**. A prestained protein ladder (PL) was used for estimation of protein size; indicated sizes are given in kDa.

The corresponding structural genes of the before identified proteins from *T. reesei*, *A. niger,* and *F. graminearum* (termed from now on Err1) were heterologously expressed in *E. coli*. Therefore, the fungi were grown on rich medium. Subsequently, RNA was isolated and reversely transcribed into cDNA, which was used as a template for amplification of the respective *err1* genes. Cloning into a pGEX vector allowed the expression as GST fusion proteins. After induction using IPTG, the *E. coli* cells were decomposed and the three GST::Err1 fusion proteins were isolated via a corresponding purification system. Soluble enzyme expression and purification of all three proteins at their correct, calculated size (64, 64, and 65 kDa, for the protein from *T. reesei, A. niger,* and *F. graminearum,* respectively) was confirmed using SDS-PAGE (Figure 2).

### Optimal parameters for the erythrose reductase enzyme assay

Since neither of the proteins has yet been characterized using D-erythrose as a substrate, the optimal parameters for the enzymatic assay had to be determined. Enzyme assays were performed using the proteins heterologously expressed in *E. coli*.

For the reducing reaction, which converts D-erythrose to erythritol, a previous study reported a pH of 7.0 for the ER from *C. magnoliae* Lee et al. ([2003]). For the GLD1 from *T. reesei* Liepins et al. ([2006]) also reported a pH optimum of 7.0, but this was determined with different substrates. Therefore, Sorenson’s phosphate buffers from pH 6.0 to 8.0 were tested in steps of 0.5 pH units. Online resource 2 depicts the measured progression of the absorption caused by NADPH consumption. We found that a pH of 6.5 is clearly favorable for the Err1 from *T. reesei* (Additional file 2a). The enzymes from *A. niger* and *F. graminearum* showed strongest decrease in absorbance at pH 7.0, but the differences between varying pH conditions were neglible for both (Additional file 2b and Additional file 2c). Therefore, the temperature optimization was carried out at pH 6.5 for all three enzymes from 10°C to 50°C (in steps of 10°C). For Err1 from *T. reesei* we found an increase in activity between 10°C and 40°C, whereas 40°C and 50°C already yielded almost identical activities (Additional file 2d). The enzyme from *A. niger* showed only slightly better performance at 50°C compared to 40°C (Additional file 2e). For the Err1 from *F. graminearum* enzyme denaturation occurred most probably at 50°C, which can be deduced from the early loss of activity at a still high NADPH concentration (Additional file 2f). Since the improvement in Err1 activity using 50°C instead of 40°C was negligibly anyway and with respect to better enzyme stability, 40°C was chosen for further measurements.

Testing the three enzymes under optimized conditions with NADH instead of NADPH as co-factor for neither of them yielded a detectable activity. This is in accordance with former reports on the *T. reseei* enzyme, which showed activity only under consumption of NADPH, but not with NADH Liepins et al. ([2006]).

For the oxidizing reaction, which converts erythritol to D-erythrose under consumption of NADP^+^, former studies proposed a pH of about 9 for similar reactions Colowick ([1963]). Consequently, Tris/HCl buffers of pH 8.0, 8.5, and 9.0 (equals the upper range of this buffer system) were tested at an assay temperature of 40°C. Only at pH 9 the oxidation of erythritol was the favored direction of the reaction, however, it proceeded much slower than the inverse reaction described before. At pH 8.5 an oscillating reaction was observed, whereas at pH 8.0 the equilibrium was completely on the reducing side of the reaction (data not shown).

Altogether, we suggest the usage of a buffers system at pH 6.5 and a temperature of 40°C for the erythrose reductase assay.

### Substrate specificity and activity of Err1

Substrates were chosen in order to cover molecules from 2 to 6 carbon atoms (C2 – C6) on the one hand, and aldehydes and ketones on the other hand: the dialdehyde glyoxal (C2), the keto-aldehyde methylglyoxal (C3), the trioses DHA and L-glyceraldehyde, the aldotetroses D-erythrose and D-threose, the aldopentoses L-arabinose and D-xylose, the ketopentose D-xylulose, and the aldohexose D-glucose.

The three enzymes showed some differences in both, substrate specificity as well as in total activity. But for all of them the activity using DHA, D-glucose, D-xylose, and D-xylulose was too low to evaluate the kinetics parameters. Consequently, these substances will be neglected in the further discussion.

The Err1 from *T. reesei* seemed to slightly favor D-threose over the other substrates, but showed only slight differences in K_m_ considering the standard deviations (Table 2). The turnover number (k_cat_) on the other hand was for methylglyoxal and L-glyceraldehyde higher than for D-erythrose, followed by D-threose in the fourth place and here the differences were considerably. Looking at the catalytic efficacy (k_cat_/K_m_), D-threose performed a little bit better than L-glyceraldehyde and D-erythrose, only to be seconded by methylglyoxal (Table 2). Altogether, the enzyme had a similar good performance for D-erythrose and D-threose and therefore, obviously here lacks stereospecificity. L-glyceraldehyde had the lowest specificity considering K_m_, but the second best k_cat_. The catalytic efficacy was about the same as for D-threose. Glyoxal had a K_m_ between D-threose and D-erythrose, but k_cat_ and catalytic efficacy were lower than for both, D-erythrose and D-threose. The same is true for L-arabinose, only that k_cat,_ and therefore also k_cat_/K_m_, was much lower (about 10-fold) than for the other substrates.

**Table 2:** **Substrate specificity of Err1 from *T. reesei***

**Substrate**^**a**^	**K**_**m**_**[μM]**	**k**_**cat**_**[kat/mol]**	**k**_**cat**_**/K**_**m**_**[1/(mM·s)]**
L-arabinose	124.56 ± 9.78^b^	3.21 ± 0.22	25.80 ± 0.23
Dihydroxyaceton	n.d.^c^	n.d.	n.d.
D-erythrose	134.52 ± 9.34	36.51 ± 2.13	271.41 ± 3.00
D-glucose	n.d.	n.d.	n.d.
L-glyceraldehyde	158.04 ± 5.00	47.89 ± 1.86	303.02 ± 2.18
Glyoxal	102.74 ± 9.76	18.84 ± 1.34	183.41 ± 4.40
Methylglyoxal	131.86 ± 1.84	72.58 ± 0.28	550.41 ± 5.55
D-threose	94.07 ± 2.46	29.03 ± 0.89	308.59 ± 1.36
D-xylose	n.d.	n.d.	n.d.
D-xylulose	n.d.	n.d.	n.d.

^a^ listed in alphabetical order.

^b^ mean of three replicates and standard deviation is given.

^c^ means not detectable.

Referring to K_m_, the *A. niger* enzyme clearly preferred D-erythrose (Table 3). On the other hand, k_cat_ and the catalytic efficacy were comparably low for D-erythrose, but very high for D-threose. With methylglyoxal the best performance was achieved, but with relatively low specificity. A similar result was found for L-glyceraldehyde, which performed second best considering k_cat_ and showed a similar K_m_. For the *A. niger* enzyme glyoxal reached a k_cat_ higher than that of D-erythrose, but with a worse K_m_, so k_cat_/K_m_ was still higher for D-erythrose. The utilization of L-arabinose led to similar kinetic parameters as obtained with the *T. reesei* enzyme.

**Table 3:** **Substrate specificity of Err1 from *A. niger***

**Substrate**^**a**^	**K**_**m**_**[μM]**	**k**_**cat**_**[kat/mol]**	**k**_**cat**_**/K**_**m**_**[1/(mM·s)]**
L-arabinose	286.66 ± 27.06^b^	7.32 ± 0.51	25.55 ± 0.64
Dihydroxyaceton	n.d.^c^	n.d.	n.d.
D-erythrose	139.39 ± 6.45	24.95 ± 1.05	179.00 ± 0.76
D-glucose	n.d.	n.d.	n.d.
L-glyceraldehyde	319.28 ± 4.12	143.23 ±1.12	448.61 ± 2.29
Glyoxal	330.95 ± 3.06	49.09 ± 0.68	148.34 ± 0.68
Methylglyoxal	352.81 ± 24.42	196.04 ± 13.43	555.66 ± 0.39
D-threose	279.50 ± 7.89	108.44 ± 1.98	387.97 ± 3.87
D-xylose	n.d.	n.d.	n.d.
D-xylulose	n.d.	n.d.	n.d.

^a^ listed in alphabetical order.

^b^ mean of three replicates and standard deviation is given.

^c^ means not detectable.

The Err1 from *F. graminearum* slightly favored methylglyoxal over D-erythrose looking at the K_m_, but again the difference was not significant (Table 4). In k_cat_ and catalytic efficacy D-erythrose was also only excelled by methylglyoxal and L-glyceraldehyde. D-threose and glyoxal had a similar turnover rate, but considering K_m_ the specificity was much higher for D-threose. For L-arabinose no measurable activity was found. Generally, it is notable that for all substrates k_cat_ and catalytic efficacy were much lower (more then 10-fold) if the *F. graminearum* enzyme was used compared to those from the other two species.

**Table 4:** **Substrate specificity of Err1 from *F. graminearum***

**Substrate**^**a**^	**K**_**m**_**[μM]**	**k**_**cat**_**[kat/mol]**	**k**_**cat**_**/K**_**m**_**[1/(mM·s)]**
L-arabinose	n.d.^b^	n.d.	n.d.
Dihydroxyaceton	n.d.	n.d.	n.d.
D-erythrose	227.61 ± 8.81^c^	3.72 ± 0.09	16.36 ± 0.23
D-glucose	n.d.	n.d.	n.d.
L-glyceraldehyde	298.72 ± 88.4	8.54 ± 2.28	28.57 ± 0.83
Glyoxal	535.16 ± 6.42	2.90 ± 0.07	5.42 ± 0.06
Methylglyoxal	214.32 ± 7.64	6.76 ± 0.24	31.55 ± 0.02
D-threose	380.48 ± 18.65	2.91 ± 0.00^d^	7.64 ± 0.37
D-xylose	n.d.	n.d.	n.d.
D-xylulose	n.d.	n.d.	n.d.

^a^ listed in alphabetical order.

^b^ means not detectable.

^c^ mean of three replicates and standard deviation is given.

^d^ means < 0.01.

### Overexpression of *err1* in *T. reesei* proves its function in vivo

**Figure 3:**
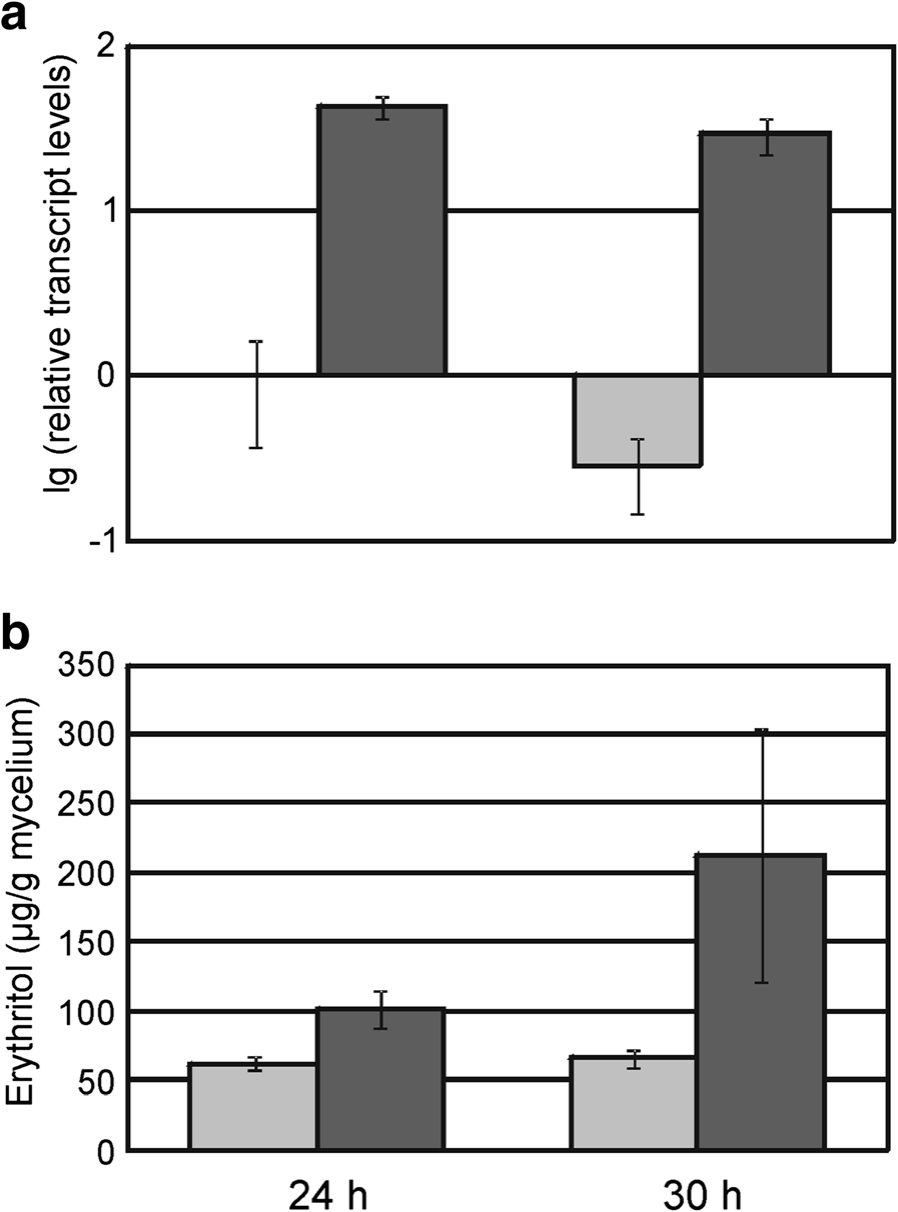
**Influence of *err1 *overexpression in *T. reesei *in vivo.** Strains were grown in duplicates on MA medium supplemented with 1% D-xylose in shakeflasks. Samples of mycelia were drawn after 24 h and 30 h. **(a)** Relative transcript levels of *err1* in the *T. reesei* parental strain (light grey) and the *err1* overexpression strain (dark grey). Transcript levels of *err1* were analysed in triplicates by RT-qPCR and normalized using *sar1* and *act1* as reference genes. All transcript levels refer to the one from the parental strain after 24 h (reference sample). **(b)** Erythritol formation in the *T. reesei* parental strain (light grey) and the *err1* overexpression strain (dark grey). Erythritol content of the mycelia was determined in triplicates by GC analysis. Error bars indicate standard deviations.

To investigate if Err1 in vivo really has the proposed functionality, an according overexpression strain of *T. reesei* was constructed and its production of erythritol was compared to its parental strain. For constant expression we have put the *T. reesei err1* gene under control of the constitutive *pki* promoter and transformed the construct into the fungal genome. The strains received from protoplast transformation were analyzed by PCR with regard to the presence of the vector construct. Positive ones were screened for *err1* expression based on transcript analysis, and the one with the highest increase in *err1* expression compared to its parental strain (named PEC1) was chosen for further characterization. Both, the parental and recombinant strain were grown on D-xylose as carbon source for 30 h in shakeflasks. Samples were drawn after 24 h and 30 h, and subsequently used for RT-qPCR and GC analysis. RT-qPCR confirmed a considerably elevated transcript level of *err1* in the recombinant strain compared to the parental strain, which was already observed during the above-mentioned screening process (Figure 3a).

GC analysis of the intracellular erythritol concentration of both strains demonstrated that the *err1* overexpression strain indeed was able to produce more erythritol than its parental strain. After 24 h, the erythritol concentration in the recombinant strain was 1.6-fold higher than in the parental strain, and after 30 h it was even 3.2-fold, respectively (Figure 3b).

## Discussion

Based on the protein sequences of the known erythrose reductases from *Trichosporonoides megachiliensis* SNG-42 (Ookura et al. [2005]), we identified by in silico analysis candidate proteins for Err1 in *T. reesei*, *A. niger,* and *F. graminearum*. In vitro analysis of these proteins by an enzyme assay confirmed for all of them a high substrate specificity and turnover rate for D-erythrose. Out of ten tested aldehydes and ketones, ranging from C2 to C6, only methylglyoxal and L-glyceraldehyde partly showed better performance or substrate specificity than D-erythrose and its diastereomer D-threose. For the cell toxin methylglyoxal it is known that aldehyde reductases show considerable activity for it, and convert it to hydroxyacetone (95%) and D-lactaldehyde (5%) (Thornalley [1996]). But the main detoxification of methylglyoxal is done by the glyoxalase system, consisting of glyoxalase I and II and catalytic amounts of reduced glutathione. These enzymes belong to superfamily cl14632, whereas Err1 belongs to superfamily cl00470 and utilizes NADPH as cofactor. Therefore, it is very unlikely that Err1 belongs to the glyoxalase system. Interestingly, the good performance of erythrose reductase with glyceraldehydes, which was observed in this study, was also reported by (Lee et al. [2003]) for *C. magnoliae*.

Neither of the tested Err1 proteins from the three fungi has a clear specificity for D-erythrose over D-threose or vice versa. In case of the Err1 from *T. reesei* D-erythrose showed a higher turnover number than D-threose, but the differences in K_m_ were not substantially. The Err1 from *A. niger* on the one hand clearly preferred D-erythrose considering K_m_, but on the other hand, the turnover number was considerably higher for D-threose. Only the enzyme from *F. graminearum* has a slight preference for D-erythrose, which is reflected by both characteristic numbers, K_m_ and k_cat_. Since Err1 takes various short-chained aldehydes as substrate it is not surprising that it utilizes the diastereomers D-erythrose and D-threose in a similar manner.

Aside from D-erythrose (C4), D-threose (C4), L-glyceraldehyde (C3) and methylglyoxal (C3) also glyoxal (C2) caused distinct activity. The enzymes from *T. reesei* and *A. niger* also showed measurable activity with the C5-sugar L-arabinose, but it was much lower than the activity of the substrates mentioned before. With D-xylose, the other C5-aldehyde tested, only a poor activity of these two enzymes was detected, which turned out to be too low to calculate kinetic parameters. The C6-sugar D-glucose showed no activity at all. It can therefore be proposed that Err1 is limited to substrates with a chain length ≤ 5 C-atoms, with best performance for 3 and 4 C-atoms. The two ketones analyzed, DHA and D-xylulose, showed no measurable activity. This leads to the assumption that only aldehydes are suitable substrates, which is in accordance with the previous general assignment of the *A. niger* enzyme as aldehyde reductase.

The Err1 from *T. reesei* and *A. niger* performed quite similar (activity is in the same order of magnitude), whereas the enzyme from *F. graminearum* showed much lower activity (about one tenth) for all substrates. Also, the latter was found to be less temperature-stable than the other ones, as the loss of activity was visible within minutes if kept at 50°C.

Comparing the kinetic parameters using D-erythrose as substrate and NADPH as co-factor, a ten times higher K_m_ was observed for the Err1 proteins from *T. reesei* and *A. niger* characterized in this study than for ER1 and ER2 from *C. magnoliae* (Lee et al. [2010]). The k_cat_ of Err1 from *T. reesei* and *A. niger* is in the same order of magnitude as ER2, resulting in a 10-fold higher catalytic efficacy of ER2. The strict requirement of NADPH as cofactor is in accordance with results for *C. magnoliae* (Lee et al. [2010]). However, the presence of erythrose reductase activity in these filamentous fungi is an important prerequisite for the possibility of developing production strategies using non-food plant biomass. Notably, the enhanced *err1* expression in a recombinant *T. reesei* strain led to an increased formation of erythritol. Even if the yield is not at the level of the yeast production strains, it should be considered that these strains have already undergone extensive mutagenesis and were screened for erythritol production. Any kind of engineering steps are still open in order to increase erythritol production in filamentous fungi. As this is an attractive alternative that would use cheap and sustainable starting materials an according patent was issued (Mach-Aigner et al. [2012]).

Finally, the recombinant *T. reesei* strain, which overexpressed *err1,* and its parental strain demonstrated functionality of the erythrose reductase in vivo. This emphasizes that the earlier characterizations of the enzyme from *T. reesei* as Gld1 (Liepins et al. [2006]) and the one from *A. niger* as Alr1 missed an important biological function of the enzyme. In summary, all three levels of investigation (in silico, in vitro, and in vivo) have provided evidence that the proteins identified are catalyzing the side reaction of the PPP, in which D-erythrose is converted to erythritol and vice versa. Altogether, this supports their capability to function as erythrose reductases.

## Competing interests

A European patent entitled “Method for the production of erythritol” (no. EP20100183799, 5.4.2012) was issued.

## Authors’ contributions

BJ participated in cloning of the genes, carried out heterolgous expression and purification of the enzymes, participated in enzyme assay optimization, generated and characterized recombinant strains, and helped to draft the manuscript. RLM drafted the concept of the study and participated in the drawing of the phylogenetic tree. ARMA participated in cloning of the genes and enzyme assay optimization, prepared the manuscript, and supervised experimental design. All authors read and approved the final manuscript.

## Supplementary Material

Additional file 1 Schematic drawing of the metabolic pathway concerning erythritol as a side product of the phosphate pathway. Click here for file

Additional file 2 **Determination of the optimal pH and temperature for assaying Err1 activity from filamentous fungi.** Collecting the absorbance data was restarted 60 s after the enzyme reaction was started by addition of D-erythrose and was continued over the time indicated in s. Different pH conditions (6.0, dark blue; 6.5, orange; 7.0, yellow; 7.5, light blue; 8.0, dark red) at 40°C (a, b, c) and different temperatures (10°C, dark blue; 20°C, orange; 30°C, yellow; 40°C, light blue; 50°C, dark red) at pH 6.5 (d, e, f) were tested using GST-fusion proteins of Err1 from *T. reesei* (a, d), *A. niger* (b, e), and *F. graminearum* (c, f). Click here for file
