# Supplementary material for: Characterization of erythrose reductases from filamentous fungi
Source: AMB Express. 2013 Aug 8;3:43. doi: 10.1186/2191-0855-3-43 (PMC3751045; doi:10.1186/2191-0855-3-43)
Supplement: Additional file 1 — Schematic drawing of the metabolic pathway concerning erythritol as a side product of the phosphate pathway. [file 2191-0855-3-43-S1.pdf]

Electronic supplementary material

Characterization of erythrose reductases from filamentous fungi

AMB Express

Birgit Jovanović, Robert L. Mach, and Astrid R. Mach-Aigner*

Department for Biotechnology and Microbiology, Institute of Chemical Engineering, Vienna University of Technology, Gumpendorfer Str. 1a, A-1060 Wien, Austria

* Corresponding author: Department for Biotechnology and Microbiology, Institute of Chemical Engineering, Vienna University of Technology, Gumpendorfer Str. 1a, A-1060 Wien, Austria, Tel.: +43 1 58801 166558, Fax: +43 1 58801 17299, E-mail: astrid.mach-aigner@tuwien.ac.at

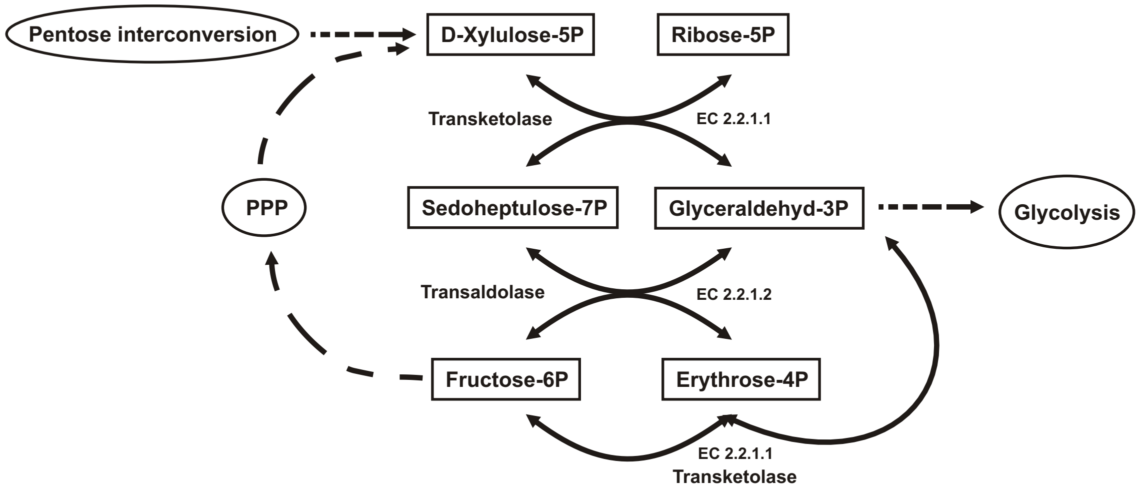

Online Resource 1: Schematic drawing of the metabolic pathway concerning erythritol as a side product of the pentose phosphate pathway.
