# Supplementary material for: Characterization of erythrose reductases from filamentous fungi
Source: AMB Express. 2013 Aug 8;3:43. doi: 10.1186/2191-0855-3-43 (PMC3751045; doi:10.1186/2191-0855-3-43)
Supplement: Additional file 2 — Determination of the optimal pH and temperature for assaying Err1 activity from filamentous fungi. Collecting the absorbance data was restarted 60 s after the enzyme reaction was started by addition of D-erythrose and was continued over the time indicated in s. Different pH conditions (6.0, dark blue; 6.5, orange; 7.0, yellow; 7.5, light blue; 8.0, dark red) at 40°C (a, b, c) and different temperatures (10°C, dark blue; 20°C, orange; 30°C, yellow; 40°C, light blue; 50°C, dark red) at pH 6.5 (d, e, f) were tested using GST-fusion proteins of Err1 from T. reesei (a, d), A. niger (b, e), and F. graminearum (c, f). [file 2191-0855-3-43-S2.pdf]

Electronic supplementary material

Characterization of erythrose reductases from filamentous fungi

AMB Express

Birgit Jovanović, Robert L. Mach, and Astrid R. Mach-Aigner*

Department for Biotechnology and Microbiology, Institute of Chemical Engineering, Vienna University of Technology, Gumpendorfer Str. 1a, A-1060 Wien, Austria

* Corresponding author: Department for Biotechnology and Microbiology, Institute of Chemical Engineering, Vienna University of Technology, Gumpendorfer Str. 1a, A-1060 Wien, Austria, Tel.: +43 1 58801 166558, Fax: +43 1 58801 17299, E-mail: astrid.mach-aigner@tuwien.ac.at

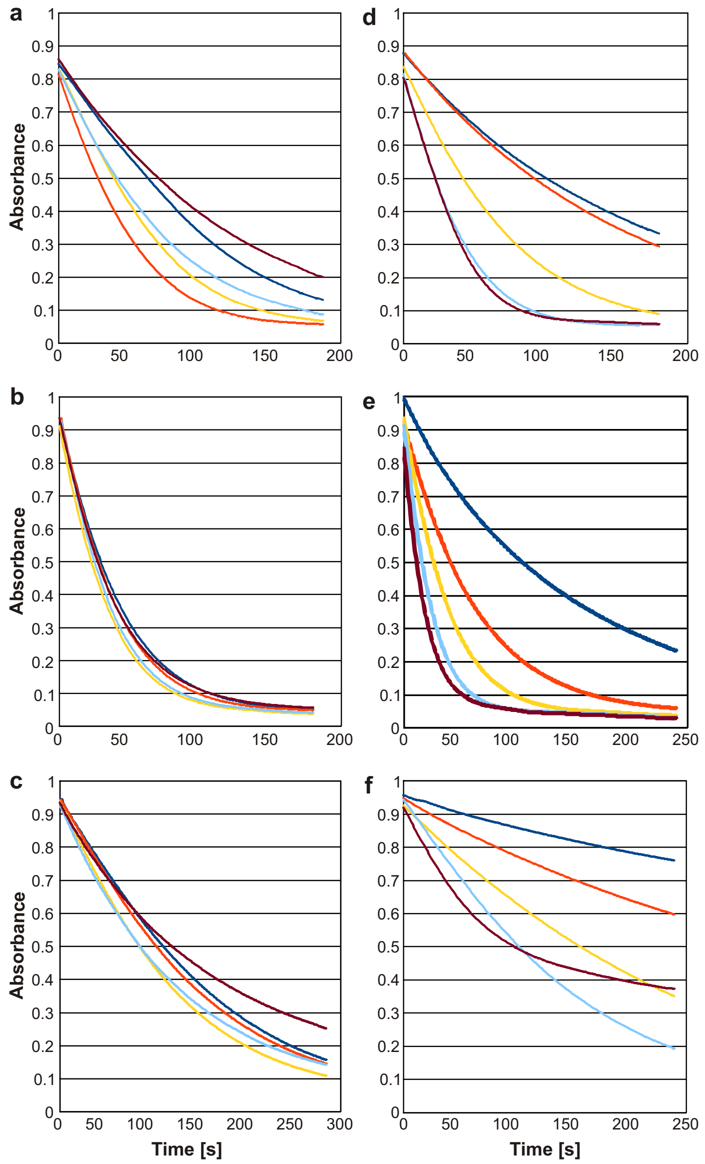

Online Resource 2: Determination of the optimal pH and temperature for assaying Err1 activity from filamentous fungi. Collecting the absorbance data was started 60 s after the enzyme reaction was started by addition of D-erythrose and was continued over the time indicated in s. Different pH conditions (6.0, dark blue; 6.5, orange; 7.0, yellow; 7.5, light blue; 8.0, dark red) at 40 °C (a, b, c) and different temperatures (10 °C, dark blue; 20 °C, orange; 30 °C, yellow; 40 °C, light blue; 50 °C, dark red) at pH 6.5 (d, e, f) were tested using GST-fusion proteins of Err1 from T. reesei (a, d), A. niger (b, e), and F. graminearum (c, f).
